# The recovery trajectory of anterior cruciate ligament ruptures in randomised controlled trials: A systematic review and meta‐analysis of operative and nonoperative treatments

**DOI:** 10.1002/ksa.12626

**Published:** 2025-02-20

**Authors:** Ali Ridha, Siddarth Raj, Henry Searle, Imran Ahmed, Nicholas Smith, Andrew Metcalfe, Chetan Khatri

**Affiliations:** ^1^ Trauma and Orthopaedics University of Warwick Coventry United Kingdom of Great Britain and Northern Ireland; ^2^ Trauma and Orthopaedics University Hospitals Coventry and Warwickshire NHS Trust Coventry United Kingdom of Great Britain and Northern Ireland; ^3^ Trauma and Orthopaedics University Hospitals Coventry and Warwickshire NHS Trust, Walsgrave General Hospital Coventry United Kingdom of Great Britain and Northern Ireland; ^4^ Trauma and Orthopaedics University Hospitals Coventry and Warwickshire NHS Trust, UHCW Coventry United Kingdom of Great Britain and Northern Ireland; ^5^ Trauma and Orthopaedics University Hospitals Coventry and Warwickshire NHS Trust, Walsgrave General Hospital, University of Warwick Coventry United Kingdom of Great Britain and Northern Ireland

**Keywords:** ACL, knee, PROMs, reconstruction, rehabilitation

## Abstract

**Purpose:**

The purpose of this research was to understand the trajectory of recovery following anterior cruciate ligament (ACL) reconstruction compared to nonoperative treatments.

**Methods:**

A systematic review and meta‐analysis approach was used to evaluate randomised controlled trials (RCTs). A comprehensive search was conducted on databases including Medline, Embase, Web of Science and The Cochrane Central Register of Controlled Trials up until 18 May 2023. The study focused on full‐text RCTs involving patients with partial or complete ACL tears. Included were studies focusing on patients undergoing ACL reconstruction or nonoperative care. The primary outcome was characterising the effects of treatments and tracking changes in International Knee Documentation Committee Subjective Knee Evaluation Form (IKDC) outcomes over time. The secondary outcome was characterising and tracking the changes of the knee injury and osteoarthritis outcome score subscales, ACL‐quality‐of‐life questionnaire, Lysholm, Tegner and CKRS scores.

**Results:**

A total of 84 RCTs were included. The pooled standardised mean changes for the IKDC compared with baseline were: 2.0 (95% confidence interval [CI]: 0.3–3.6) at 3 months, 2.2 (95% CI: 0.9–3.6) at 6 months, 2.2 (95% CI: 0.8–3.6) at 12 months and 2.3 (95% CI: 1.3–3.4) at 24 months. Graphs illustrating IKDC scores over time further emphasise these findings, showing a sustained improvement over time to 12 months, with a plateauing of scores past this time point. Our secondary outcome patient‐reported outcome measures (PROMs) also showed a similar pattern with scores plateauing at the 12‐months mark.

**Conclusion:**

Our findings suggest that the IKDC score and other PROMs are effective for tracking recovery up to 12 months. Other PROMs show pain and daily activities generally recover within 6 months, and quality of life improves up to 12 months, but PROMs show minimal improvement beyond this period. This inconsistency with a return sport period indicates that PROMs may lack the sensitivity required to assess this aspect of recovery accurately.

**Level of Evidence:**

Level I.

**Abbreviations**
ACL: anterior cruciate ligament
ACL QOL: anterior cruciate ligament quality‐of‐life questionnaire
CKRS: Cincinnati Knee Rating System
IKDC: International Knee Documentation Committee Subjective Knee Evaluation Form
KOOS: knee injury and osteoarthritis outcome score subscales
MTT: modern test theory
PROMs: patient‐reported outcome measures
RCT: randomised controlled trials
SMC: standardised mean change

## INTRODUCTION

There are three used treatment methods for people who have ruptured their anterior cruciate ligament (ACL): nonoperative management (typically rehabilitation/physiotherapy), repair and reconstruction [31]. Repair is relatively uncommon, and surgical reconstruction often becomes the chosen approach for younger and more athletically active individuals, given its potential to reduce ongoing instability, return athletes to their chosen sports and potentially to mitigate further injuries to the meniscus and cartilage [35, 36]. In the United Kingdom, there is currently over 10,000 ACL reconstructions occurring every year [1].

In other musculoskeletal conditions, such as meniscal tears [3], lower back pain [4, 5] and rotator cuff injuries [20], there is a sustained pattern of improvement in outcomes over time for participants irrespective of treatment offered. The improvement observed in certain situations can be due to various reasons, with ‘regression to the mean’ being a common explanation. This term describes how extreme starting points often balance out to be closer to the average over time [19]. This consistent improvement in outcomes can be viewed as part of the progression or trajectory of the condition and can be hard to distinguish from the effect of a treatment.

In addition, many papers that measure between intervention and control or placebo assume that any change in scores over time is a result of an intervention and the trajectory of the condition is not often considered.

Understanding the expected recovery outcomes for different treatment approaches is crucial for informing clinical decisions and setting patient expectations. This study aims to review the trajectory of recovery following ACL rupture, whether treated operatively or nonoperatively, by examining existing studies to provide clinicians with insights into the timeframes in which patients can expect improvements according to patient‐reported outcome measures (PROMs). Additionally, we will assess the performance of PROMs in capturing these recovery trajectories over time. Furthermore, we hypothesise that the natural improvement in symptoms observed over time in both groups will reflect a regression to the mean, potentially influencing the perceived effectiveness of the treatments.

## METHODS

The study was reported in accordance with the PRISMA statement for reporting systematic reviews. The systematic review protocol was predefined and can be found at https//www.crd.york.ac.uk/prospero (CRD42023427102).

### Eligibility criteria

#### Study type

Only randomised controlled trials (RCTs) where included in this study as they have standardised, strict, follow‐up routines and accommodate multiple trial arms.

#### Inclusion criteria

I. Full‐text RCTs in patients who have a partial or complete tear of the ACL treated with ACL reconstruction or nonoperative care. To ensure comprehensive analysis, this review incorporates patients who undergo surgical reconstruction who also have concurrent meniscal injuries.
II. Studies reporting at least one established Knee‐related PROM (International Knee Documentation Committee (IKDC) Subjective Knee Evaluation Form [IKDC]‐SKF, Lysholm knee score, Tegner activity scale, knee injury and osteoarthritis outcome score subscales [KOOS], CKSRS, ACL‐quality‐of‐life questionnaire [QOL]) scores at baseline and for at least 1 year with exact time points.
III. English language studies only.
IV. Studies where patients had achieved skeletal maturity

#### Exclusion criteria

I. Studies not reporting any established Knee PROM.
II. Studies reporting outcomes in patients with additional major knee ligament injury or fractures around the knee. (Not including meniscal injuries.)
III. Multiple ligament injury (two or more ligaments requiring surgery).
IV. Inflammatory arthropathy.
V. Studies without a predefined follow‐up period.
VI. Abstract or conference publications.
VII. Studies including patients with revision ACL reconstruction.
VIII. Studies that only focus particular subgroups of disease that may have worse results, rather than typical ACL rupture populations.

### Outcome measures

The primary outcome measure was the IKDC, comparing baseline to 24 months using standardised mean change (SMC). The IKDC score has been shown to have advantages over other PROMs such as KOOS and Lysholm in terms of content validity, relevance of questions, and ceiling effect [27].

The secondary outcome measures included

I. KOOS at all time points and the SMC for each subscale to 24 months [34].
II. ACL‐QOL at all time points and the SMC for each subscale to 24 months [28].
III. Lysholm knee score at all time points [23].
IV. Tegner activity scale at all time points [39].
V. Cincinnati Knee Rating System (CKRS) or Modified version at all time points [30].

### Search strategy and quality assessment

We conducted comprehensive searches of Medline, Embase, Web of Science and The Cochrane Central Register of Controlled Trials, covering their inception up until 18 May 2023. All retrieved records were imported into Rayyan (http://rayyan.qcri.org). Search terms used are included in the Supporting Information materials.

After eliminating duplicate citations, titles and abstracts were screened based on predefined criteria. Two authors (A. R. and S. R.) independently evaluated each paper. In cases of discrepancies, discussions were held with senior authors (C. K. and I. A.) to reach a consensus. Following the initial screening, full‐text reviews were undertaken. Protocols screened during the initial abstract phase were revisited to check if a subsequent publication resulted from the trial, and these were included for review if applicable. We also examined the bibliographies of included studies and included any that met our inclusion criteria.

### Extracting data

We extracted outcome data from each study according to the follow‐up time period provided.

Data were extracted regarding the number of patients in each study arm, the type of intervention (defined as ACL reconstruction with or without additional interventions, rehabilitation or rehabilitation with delayed reconstruction), as well as the mean and standard deviation of the IKDC‐SKF and other secondary PROMs in addition, any other knee‐related PROM was also collected. Data on gender, mean age and the assessment time point were gathered.

If a study did not present mean or standard deviation, we estimated these values using other provided data, such as confidence intervals (CI) or *p* values, as guided by the Cochrane handbook. For studies that did not present data numerically, we attempted to contact the authors for the specific values. In instances where no response was received, we estimated values from the study's graphical representations as per previously published methods [4, 20], However, if it was difficult to interpret the graphical representation through this method (e.g., graphs were not sufficiently clear to collect reliable data) then the values were not used.

When only medians were provided and no feedback was received from the authors regarding means or standard deviations, we utilised the medians in the graphical representation but did not include these papers in the meta‐analysis.

Baseline data were captured as postinjury scores. In cases where postinjury scores were not available, but preinjury levels were, the latter was used as the baseline score.

For studies that were follow‐ups of earlier research, we included the most extended study. Any data from a preceding study on the same cohort was consolidated into a single data row to facilitate graphical representation and meta‐analysis. If discrepancies in baseline results emerged due to dropouts, the baseline from the initial study was used, as these represented the most complete data.

Attempts were made to contact authors for additional information if further information was required about study design to confirm inclusion or if there was missing data for unreported or partially unreported outcomes.

### Statistical analysis

Using R statistical software, outcome scores were plotted over time to illustrate changes from baseline through all reported follow‐up points in all treatment arms of the included studies. This visual representation facilitated a descriptive analysis of the data. In the majority of studies, the follow‐up periods extended up to 24 months.

To understand the variations in the magnitude of response across time, we utilised the SMC [29] to evaluate outcome scores at intervals of 3, 6, 12 and 24 months. Though data at other intervals existed, our emphasis was on the most frequently reported timeframes, as would be useful for designing future trials. The efficacy of this technique is grounded in its ability to depict results in terms of a continuous measurement, as evidenced in various studies [20]. A pertinent illustration of the SMC's application is its use in a research study on a novel physical therapy regimen designed for enhancing knee range of motion postsurgery. In this context, the SMC was computed by determining the mean change in range of motion, subsequently divided by the standard deviation of the change scores. Such a method effectively standardises the therapy's impact. A substantially positive SMC signified superior efficacy of the new regimen in comparison to the conventional one, while a negative SMC would suggest the opposite.

In line with methods used in other meta‐analyses [4, 20], one arm was randomly selected from each trial for analysis for those studies that have Reconstruction versus Reconstruction or Rehab versus Rehab as their study design; for the Reconstruction versus Rehab or Optional ACL Reconstruction, both trial arms where included due the paucity of data regarding rehabilitation.

Our focus was on tracking changes in outcomes over time, not on comparisons between different arms. The primary goal of this review was to characterise the outcome trajectory for treatments, rather than to determine effect sizes between intervention groups.

We computed a pooled estimate of the SMC for each time point, employing a random effects model. Studies were then categorised based on the treatment administered: reconstructive methods or nonoperative management. Those receiving reconstructive approaches combined with any additional treatments, such as specialised rehabilitation, bone marrow techniques or novel screw applications, were also grouped under the ‘reconstructive’ category. All analyses were performed in R using the ‘metafor’ package. The code for analysis is available at https://github.com/AliRidha1994/RecoveryTrajectoryAclRuptures.

### Risk of bias

Each included paper underwent a qualitative risk of bias assessment in line with Cochrane guidelines, ensuring standardised and transparent evaluation of study quality to enhance the credibility and reliability of evidence synthesis. If the primary paper did not offer enough details for a thorough risk of bias evaluation, we consulted the respective published protocols where available.

## RESULTS

**Figure 1:**
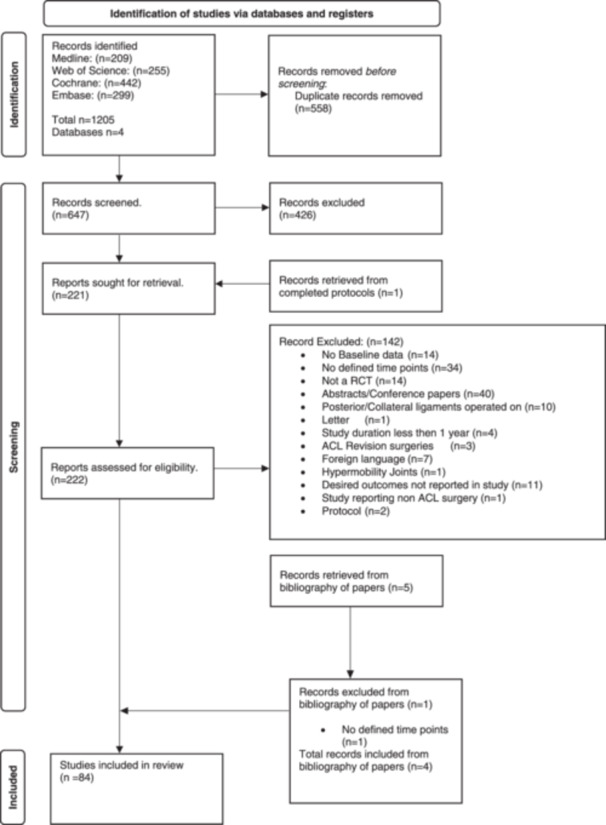
PRISMA flow diagram. This diagram provides an overview of the systematic review process, illustrating the number of papers screened, reviewed and ultimately included in the final study. It captures each stage of the review, from initial database search results to the final inclusion of relevant studies.

A total of 84 studies were included in our review, following the screening of 1205 initial citations, the removal of duplicates, and the addition of relevant studies from protocols and methodology sections (Figure 1).

We reached out to 20 authors seeking either further clarification on study details or specific data. Four responded, and their details can be found in Supporting Information material 2.

Studies that met our criteria but lacked the necessary data were excluded from our data collection unless there was successful communication with the authors. Studies that did not provide the standard deviation or those where it couldn't be calculated were not incorporated into the meta‐analysis data but were included in our graphical representation.

A breakdown of the number of studies that used each PROM is described in the Supporting Information material.

### Description of studies included

Of the 84 studies included for extraction, 16 studies featured the same study group. Consequently, these studies were consolidated during data collection for their respective PROMs, resulting in 68 unique study groups available for inclusion. Three of these 68 studies were excluded since PROM data was unavailable at the time of our review. Therefore, 65 studies were available. Detailed characteristics of all studies are presented in the Supporting Information tables.

### Description of patient population

Of the 65 unique study groups available for inclusion, we did not collect demographic data from three studies due to the unavailability of demographic data at the time [13, 22, 37], leaving us with demographic data from 62 studies.

In the 62 studies that reported demographic data, one study reporting of sex did not match the final total [10], so sex data from that study were excluded. Two studies did not provide data on sex [16, 26]. In eight studies that presented medians ages for each trial arm, we used the mean of these medians or reported the median of the collective study [2, 7, 12, 14, 15, 18, 32, 38, 41].

Full study characteristics can be found in the Supporting Information material.

### Risk of bias assessment

The risk of bias assessments undertaken for the studies that were included is available in our Supporting Information material.

### IKDC

**Figure 2:**
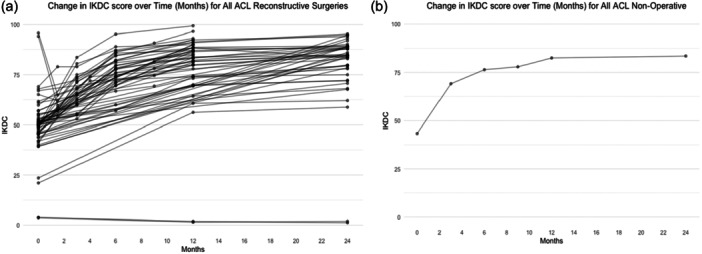
(a and b) Change in International Knee Documentation Committee (IKDC) scores over time (months) for reconstructive surgeries and nonoperative. This figures demonstrates the progression of IKDC scores over time for participants undergoing reconstructive surgeries and those receiving nonoperative treatments. The x‐axis represents time points in months, while the y‐axis displays IKDC scores on this scale, 0 indicates extreme knee problems, and 100 indicates no knee problems. Data points at each time interval are depicted as circled dots, highlighting the collated scores across all studies included in the analysis.

Overall, there was a noticeable improvement across all study arms compared to the baseline (Figure 2), particularly in studies that reported the IKDC scores. One study showed a considerably low IKDC score at all time points and is clearly seen in the graphical representation [32]. Another study used a preinjury IKDC score which reflects the initial decline from baseline to 3 months graphically and is also represented with a negative SMC on the forest plots.

There was one study that reported nonoperative treatment and IKDC scores, which displayed a positive trend of improvement over time [33]. This same study also had 41 patients that opted for ACL reconstruction included in their rehab ± treatment arm. However, this treatment arm is not displayed in our results as we were unable to obtain specific data on that group.

There is a considerable improvement from 0‐ to 12‐months range with a plateau at 24 months for the operative arms. The one nonoperative arm also shows a plateau from 12 to 24 months with 12‐months IKDC being 74.4 and 24 months being 79.4.

**Figure 3:**
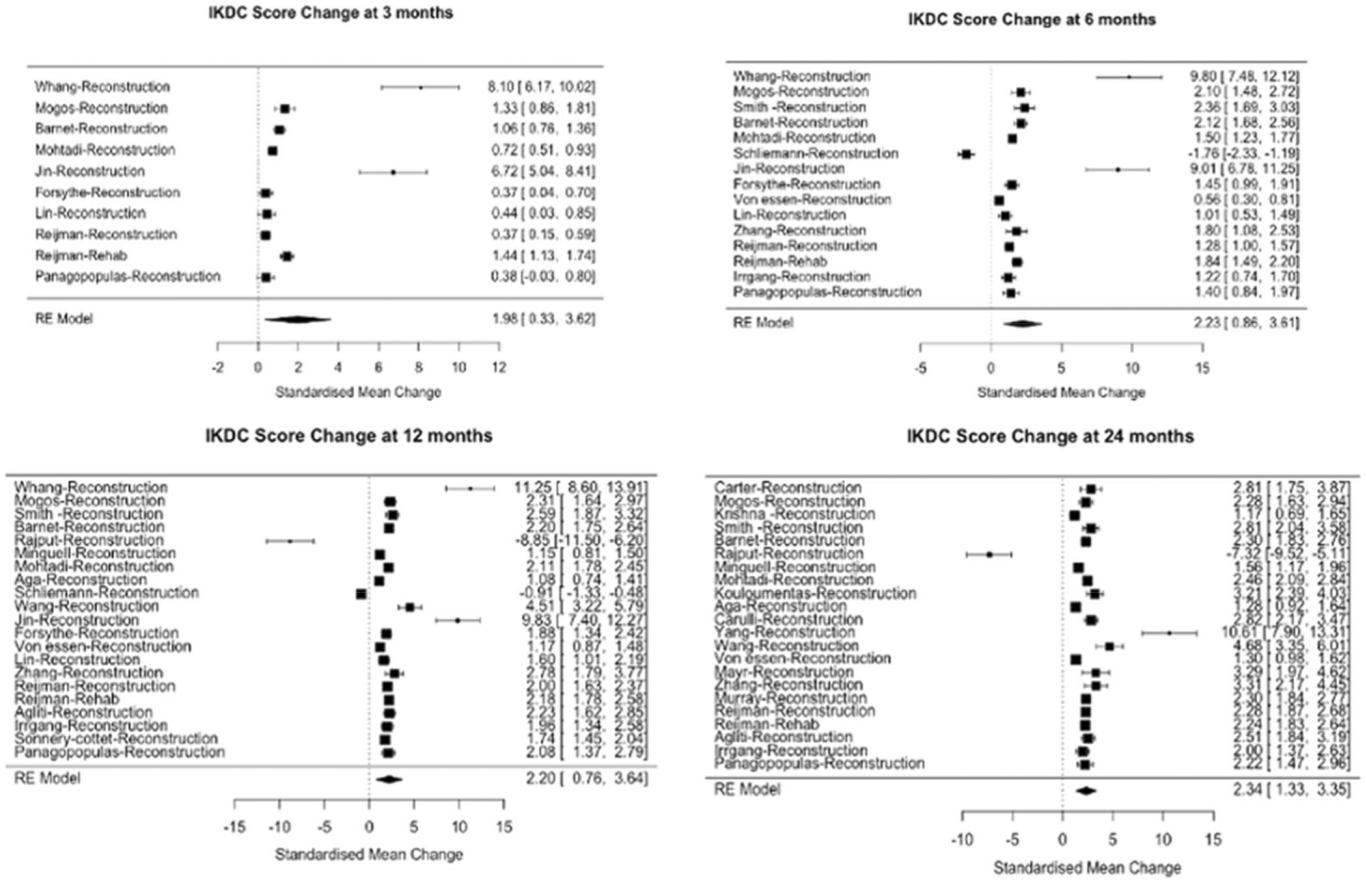
International Knee Documentation Committee (IKDC) Pooled standardised mean change (SMC) score changes over 3, 6, 12 and 24 months. This figure displays the SMC in IKDC scores for each individual trial, along with the pooled SMC scores across all included studies, at four key time points: 3, 6, 12 and 24 months. Both the reconstructive surgery and nonoperative treatment arms are included in the meta‐analysis. Each SMC value is accompanied by 95% confidence intervals, providing an indication of the variability and precision of the estimates. The pooled SMC value for each time point is located at the bottom of the corresponding forest plot, offering a comprehensive summary of knee function improvement over time.

A forest plot depicting the pooled SMC from baseline for different treatment arms was generated for the IKDC score (Figure 3), illustrating a considerable pooled treatment response. As seen in Figure 3, there is only one trial that included a rehab arm, forest plots without the rehab arm are available in the Supporting Information material.

The SMC between baseline and 3 months was 2.0 (95% CI: 0.3– 3.6), indicating a positive change in the measured variable. The CI ranged from a small increase (near zero as the lower bound) to a potentially significant increase (up to 3.6 standard deviations as the upper bound), underscoring the variability and uncertainty in the magnitude of change between these time points.

At the 6‐months mark, a positive change was evident, with an SMC of 2.2 (95% CI: 0.9–3.6), signifying a notable increase in the variable. This positive change plateaued after 6 months, with an SMC from baseline to 12 months, 2.2 (95% CI: 0.8–3.6). At 24 months, there was a further small increase, with an SMC of 2.3 (95% CI: 1.3–3.4). The rehab arm also demonstrated consistent improvement aligned with the pooled results.

There was a consistent correlation observed in IKDC SMCs across different time points as the studies progressed. The Pearson correlation coefficients were 0.9 (*n* = 12, 95% CI 0.7–1.0) between 3 and 6 months, 0.9 (*n* = 17, 95% CI 0.8–1.0) between 6 and 12 months and 1.0 (*n* = 16, 95% CI 1.0–1.0) between 12 and 24 months.

### KOOS

The KOOS4 score was calculated from the four subsections (pain, symptoms, quality of life and sport) in each trial that reported them. Both operative and nonoperative arms were plotted up to 24 months. In the operative group, the majority of KOOS4 scores peaked at 12 months and plateaued at 24 months. In the three nonoperative arms, we see a steady incline in all trial arms. The KOOS4 graphs are in the Supporting Information material. The KOOS5 score was also calculated from the five subsections in each trial in the operative group. The KOOS5 graphs are located in the Supporting Information material.

**Figure 4:**
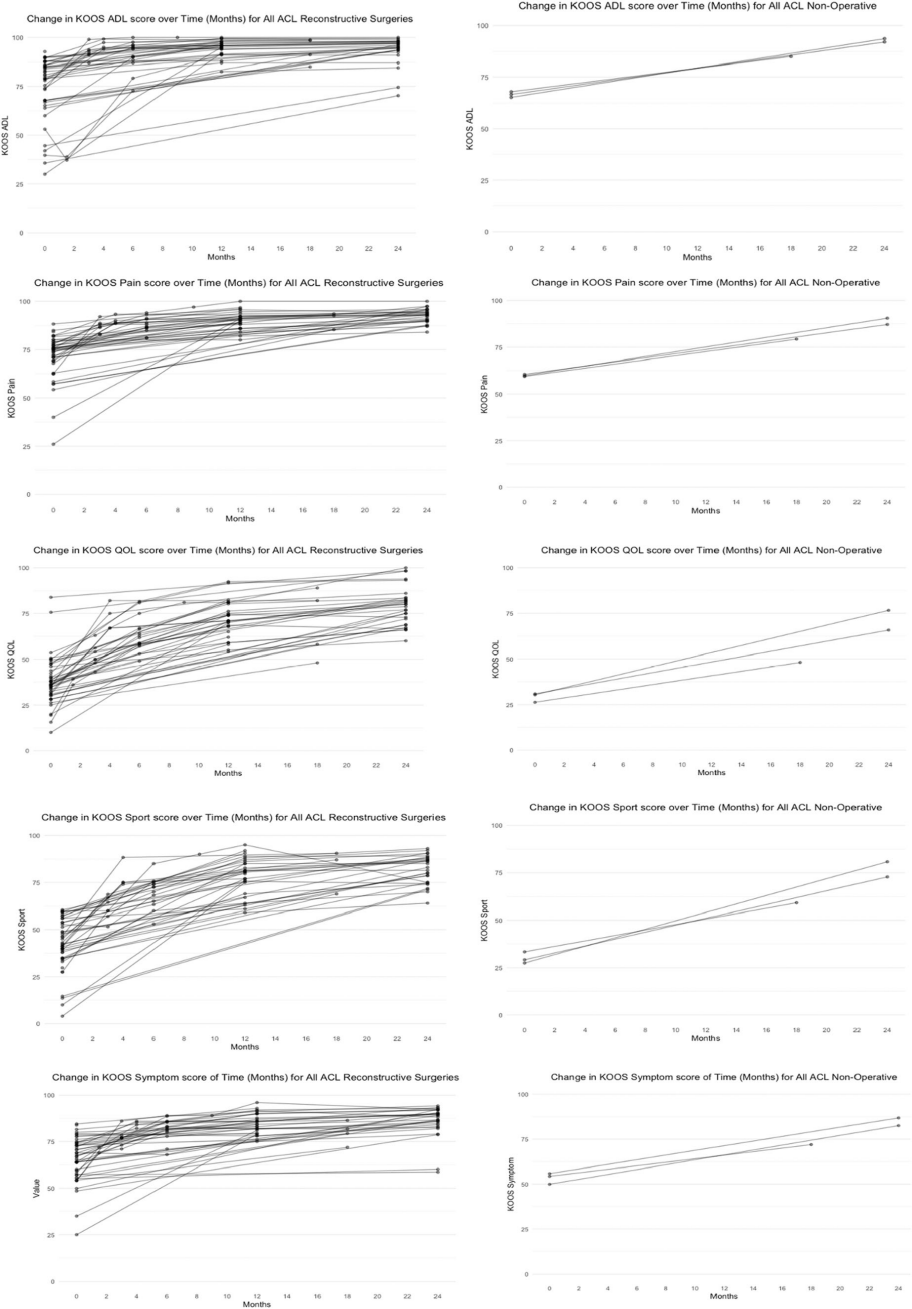
Change in Knee Injury and Osteoarthritis Outcome Score subscales (KOOS) scores over time (months) for reconstructive surgeries and nonoperative. This figure illustrates the variation in KOOS subscale scores (pain, ADL, sport, quality‐of‐life questionnaire, symptom) over time for patients undergoing reconstructive surgeries and those receiving nonoperative treatment. The x‐axis represents time in months, while the y‐axis displays KOOS subscale scores, which are transformed to a 0–100 scale. On this scale, 0 indicates extreme knee problems, and 100 indicates no knee problems. Data points at each time interval are depicted as circled dots, representing collated scores across all included studies. This provides a clear visualisation of changes in different dimensions of knee health over time. ADL, activites of daily living.

All KOOS Subscale graphs are included in Figure 4. The KOOS subscale activites of daily living (ADL) score plateaued from 6 to 24 months for the operative arms, for nonoperative studies there was not a substantial difference between 18 and 24 months scores.

The KOOS Symptom scores show a similar pattern to the KOOS ADL. Scores plateaued from 6 to 24 months for the operative arms; for nonoperative studies, there was not a substantial difference between 18 and 24 months scores.

The KOOS Pain score also follows a similar pattern in the operative arms with pain scores plateauing after 6 months, for nonoperative studies, there was not a substantial difference between 18 and 24 months scores.

The KOOS QOL scores for the operative arms plateau at 12 months; for the nonoperative arms, there is a steady improvement in all three trial arms.

The KOOS Sport scores for the operative arms plateau at 12 months; for the nonoperative arms, there is a steady improvement in all three trial arms.

The KOOS subscale SMC scores were calculated. Due to the availability of only the subscales' standard deviations, we were restricted to calculating the SMC for each individual subscale instead of an overall average. In terms of the KOOS data availability, three studies reported scores at 3 months, seven at 6 months, 10 at 12 months and 14 at 24 months. The SMC scores are presented in Table 1 alongside their respective CIs for each subscale.

**Table 1:** The SMC changes for each KOOS subscale at 3, 6, 12 and 24 months along with their respective 95% confidence interval.

KOOS ADL (months)	
3	0.5 (95% CI: −0.1,1.0)
6	1.0 (95% CI: 0.3,1.7)
12	1.0 (95% CI: 0.5,1.5)
24 (Pooled)	1.2 (95% CI:0.7,1.7)
KOOS pain (months)	
3	0.9 (95% CI: 0.6,1.2)
6s	1.2 (95% CI: 0.3,2.1)
12	1.3 (95% CI: 0.5,2.1)
24 (Pooled)	1.3 (95% CI: 0.9,1.8)
KOOS QOL (months)	
3	0.7 (95% CI: 0.2,1.1)
6	1.9 (95% CI: 0.7,3.1)
12	2.4 (95% CI: 1.2, 3.5)
24 (Pooled)	2.2 (95% CI:1.8, 2.6)
KOOS sport (months)	
3	0.6 (95% CI: 0.3,0.9)
6	1.4 (95% CI: 0.6,2.1)
12	1.7 (95% CI: 1.0,2.4)
24 (Pooled)	2.0 (95% CI: 1.5, 2.6)
KOOS symptom (months)	
3	0.5 (95% CI: 0.2,0.8)
6	1.2 (95% CI: 0.6,1.8)
12	1.2 (95% CI: 0.6,1.8)
24 (Pooled)	1.6 (95% CI: 0.8,2.4)

Abbreviations: ADL, activites of daily living; KOOS, Knee Injury and Osteoarthritis Outcome Score subscales; QOL, quality‐of‐life questionnaire; SMC, standardised mean change.

As with the IKDC, we retained both arms of studies comparing Reconstruction versus Rehab ± optional Reconstruction. Both studies had patients in their Rehab ± optional Reconstruction arms that opted for reconstruction. The COMPARE [33] study had 41 and the KANON [11] study had 23 patients over the 2‐year period that opted for reconstruction, however, only the KANON study provided a breakdown of KOOS scores for the two trial arms and the additional Rehab ± optional Reconstruction arm. The breakdown SMC scores for these subgroups are included in the Supporting Information material. Additionally, for our third comparative trial ACL SNAPP, we also included an SMC score at 18 months in the Supporting Information material.

### ACL QOL

The ACL‐QOL score also showed a similar pattern to our other PROM. The operative arms of the study increased progressively to 12 months with some studies showing some improvement from 12 to 24 months; however, the majority subsequently plateaued. There was only one nonoperative trial that used ACL‐QOL which showed a linear trajectory at 18 months. All ACL‐QOL graphs are located in the Supporting Information material.

In terms of the ACL‐QOL data availability for SMC, one study reported scores at 3 months, one at 6 months, three at 12 months and four at 24 months. The SMC scores are presented in Table 2 alongside their respective CIs for each subscale. Like the KOOS scores for our third comparative trial ACL SNAPP [6] we also included an SMC score at 18 months in the Supporting Information material. The ACL QOL SMC scores show a marked improvement like other PROM scores till 12 months but a relative plateau from 12 to 24 months is also demonstrated in the SMC.

**Table 2:** The SMC changes for each ACL QOL at 3, 6, 12 and 24 months along with their respective 95% confidence interval.

ACL QOL (months)	
3	1.6 (95% CI: 1.3,1.9)
6	2.6 (95% CI: 2.2,3.0)
12	6.9 (95% CI: 3.3,10.6)
24	6.5 (95% CI: 2.1,9.7)

Abbreviations: ACL, anterior cruciate ligament; QOL, quality‐of‐life questionnaire; SMC, standardised mean change.

### Lysholm

In terms of graphical treatment responses graphs, positive advancements were observed in Lysholm across the trial arms. The Lysholm score, like the IKDC also appear to have a levelling off after 6 months for the operative and nonoperative arms. The Lysholm graphs are also located in the Supporting Information material.

### Tegner

The pattern of change in Tegner scores was much more variable across studies. Change over time in the Tegner score might be influenced by the utilisation of preinjury scores, a detail that was not consistently mentioned in the selected studies. Studies that did refer to preinjury Tegner scores and preoperative Tegner scores were plotted on separate graphs. The Tegner scores exhibited a decline over time in the nonoperative trial arms, all arms reported a preoperative baseline score. With regard to the operative arms, there was a mixed picture with a wide variety of baseline scores and follow‐up scores. The preoperative scores stabilise at 12 months, however, some studies show a continued improvement from 12 to 24 months. The preinjury scores show a mixed picture, with some studies showing return to preinjury scores while others did not at 12–24 months. This indicates that the initial notable gains tend to level off after this period and return to higher levels of sport may be more difficult in the long term. The Tegner score covers a broad range of sporting abilities, so this may naturally change due to changes in sporting engagement or as a sporting career progresses. All Tegner graphs are in the Supporting Information material.

Due to limited data, the CKRS or Modified version where not analysed.

## DISCUSSION

The most important finding of this study is that PROMs, such as the IKDC score, are particularly useful for tracking recovery up to the 12‐months mark, with significant improvements observed across various treatment modalities during this period. Recovery of pain and daily activities typically occurs within 6 months, and quality of life continues to improve up to 12 months. However, PROMs show minimal improvement beyond this point, and the Tegner scores in the nonoperative group remain an exception. Although return to sport is often recommended between 9 and 15 months postinjury, many PROMs plateau by 12 months, suggesting they may provide limited additional insight into higher‐level recovery. The inconsistency in return to sport between 12 and 24 months further implies that PROMs may lack the sensitivity needed to accurately assess this outcome.

The IKDC scores, for instance, demonstrated a consistent positive trend starting from 3 months, with improvements plateauing between 6 and 24 months across various operative treatments. This suggests that while PROMs like the IKDC are valuable for assessing initial recovery, their sensitivity to changes in recovery beyond 12 months may be limited.

The KOOS scores also showed a similar pattern to the IKDC scores. The KOOS4 operative scores plateau at the 12‐months mark. Pain, ADL and QOL subsections show little change after the 6 months mark which may indicate that full recovery is made in these domains early or that they have limited sensitivity past 6–12 months. However, sports and symptom scores continue to have some substantial improvements at 12 months, consistent with people returning to sport over that phase.

This data supports the views of the originator of the score of the value of evaluating KOOS scores by domain and highlights the need for a more granular approach to assessing treatment efficacy [34]. It may also prompt a re‐evaluation of the pooled KOOS4 or KOOS5 instruments' sensitivity in capturing the full spectrum of patient recovery and the true impact of clinical interventions.

One previous study has also analysed the use of the KOOS in ACL reconstruction and modified it to create a targeted version for younger populations, the KOOS‐ACL [25]. This modification is particularly relevant considering the KOOS‐ACL's focused assessment of function and sport. Such focus aligns with our findings that substantial improvements in sports‐related capacities occur around the 12‐months mark, a critical phase for returning to sports activities.

It is noteworthy that the most substantial graphical improvements in most PROMs scores occurred within the 0–12‐months interval, regardless of the intervention used. However, it's important to acknowledge that there was significant crossover from the rehabilitation to the operative arms in the trials, which could influence the interpretation of these scores. Nevertheless, where possible, scores were analysed on a per‐protocol basis for these groups, and this is demonstrated in the results section.

Regarding the Tegner scores, trend in activity over time was discerned within the three nonoperative trial arms. It is also noteworthy that the studies with preoperative levels (baseline score) demonstrated a similar plateau to other PROMs from 12 to 24 months. It is also notable that in studies that measured preinjury scores, there was not a substantial change between the 12 and 24 months which is the time period that the majority of rehabilitation protocols expect return to sport [8] and graft maturation [40]. It is also important to note that Tegner scores were higher in the operative group overall as opposed to nonoperatively. Ultimately, the aim of ACL intervention in patients is return to sport which may only be demonstrated in the Tegner scores.

It's also essential to critically evaluate the validity of PROMs used in RCTs when studying the natural progression of musculoskeletal diseases. While these PROMs are frequently cited in research on ACL studies, there are concerns whether they are adequate or appropriate [17, 21].

Current PROM instruments have been developed, validated and are currently deployed using classical test theory, and this may result in scores being insufficiently sensitive to detect change especially at higher‐level function such as mid‐term recovery after ACL reconstruction [9]. The application of contemporaneous psychometric validation, using modern test theory (MTT) is limited for people with ACL injuries. Some efforts have been to apply MTT to PROM instruments such as the KOOS, to create an instrument specific to ACL injuries [25], with external validation showing improved structural validity [24]. Similar work using MTT to optimise existing instruments may provide more sensitive instruments to detect change.

Our study is a comprehensive systematic review concerning ACL tears, showing a multitude of RCTs involving ACL reconstruction with alternative techniques. It successfully illustrated the overarching trend in PROMs irrespective of reconstruction types and rehabilitation methods.

The paucity of studies centred on rehabilitation as a trial arm was a limitation. Additionally, patients that were in the rehabilitation arm of the study could opt for reconstruction. The ambiguity around whether these participants were integrated into the mean scores for the rehabilitation category or not could skew our study's conclusions. In addition, the inclusion of both partial and complete tears in our operative and nonoperative groups may influence the overall PROM score outcomes. We acknowledge that partial and complete tears might represent distinct patient populations. However, our primary objective was to evaluate the recovery trajectory following treatment, and as the treatment protocol remained consistent across the underlying diagnoses, we made the pragmatic decision to analyse both groups together.

Future review studies could consider the inclusion of non‐RCTs that follow‐up those patients within a nonoperative arm. In addition, we did not analyse the trends for those in differing age groups undergoing reconstruction or rehabilitation. Furthermore, we investigated PROMs which may not satisfy the complete picture of what an individual participant is interested in such as return to specific sports, rerupture rates, or the risk of other knee injuries.

For clinicians, this highlights the importance of setting realistic expectations around sport‐specific recovery, while recognising that PROMs remain useful tools for tracking broader aspects of functional recovery. Clinicians should consider supplementing PROMs with measures better suited to assess long‐term athletic recovery in ACL patients.

## CONCLUSIONS

This review underscores that while PROMs are valuable for assessing overall recovery in patients with ACL ruptures within the first s, they may be less effective for capturing sport‐specific recovery outcomes beyond this point. Recovery in pain and daily activities generally stabilises within 6 months, and improvements in quality of life are typically seen up to 12‐months postinjury. However, beyond 12 months, current PROM instruments show limited sensitivity in evaluating higher‐level athletic recovery, as indicated by the plateau in scores and inconsistent return‐to‐sport rates between 12 and 24 months. Clinicians should set realistic expectations for sport‐specific recovery while using PROMs to track overall functional progress.

This table presents the SMC in KOOS subscale scores at four key time points: 3, 6, 12 and 24 months. A pooled analysis was conducted only at the 24‐months time point due to the availability of nonoperative studies reporting KOOS scores at that interval. The meta‐analysis incorporates data from both reconstructive surgery and nonoperative treatment arms. Each SMC value is accompanied by 95% CIs, offering insight into the variability and precision of the reported estimates.

This table presents the SMC in ACL QOL scores at four key time points: 3, 6, 12 and 24 months. The data is exclusively from the reconstructive surgery arms, as the nonoperative trial that used this PROM reported data only at baseline and 18 months. The 18‐months time point data is not included in this table but is provided in the Supporting Information files. Each SMC value is accompanied by 95% CIs, offering insights into the variability and precision of the reported estimates.

## AUTHOR CONTRIBUTIONS

All authors conceptualised the work and the design of the study. Siddarth Raj, Ali Ridha, Chetan Khatri and Imran Ahmed performed the initial searches and extraction of data. Henry Searle and Siddarth Raj performed the risk of bias. Henry Searle and Ali Ridha performed the statistical analysis of the data. Chetan Khatri, Imran Ahmed, Andrew Metcalfe and Nicholas Smith contributed to the interpretation of the data and its application in the wider research field. All authors contributed to the initial drafting and subsequent revisions of the article.

## CONFLICT OF INTEREST STATEMENT

Mr. Nicholas Smith: Arthrex, fee for presenting at OTIF conference Munich and reimbursement for attending OTIF conference Naples. Andrew Metcalfe is an investigator on three NIHR‐funded trials (START:REACTS, RACER‐Knee and RACER‐Hip, Andy Metcalfe leads START:REACTS and RACER‐Knee), for which Stryker also fund treatment costs and some imaging costs. For all these studies, the full independence of the study team is protected by legal agreements. Mr. Chetan Khatri: Received a salary as a clinical research fellow as a part of the RACER Knee trials. As a part of the RACER Knee trial, Stryker, a medical device company, fund some treatment costs and imaging costs. This manuscript received no funding from Stryker. The independence of the research is protected by legal agreements and have no bearing on the presented study. The remaining authors declare no conflicts of interest.

## ETHICS STATEMENT

The authors have nothing to report.

## Supporting information

Supporting information.

Supporting information.

Supporting information.

Supporting information.

Supporting information.

Supporting information.

Supporting information.

Supporting information.

Supporting information.

Supporting information.

Supporting information.

Supporting information.

Supporting information.

Supporting information.

Supporting information.

Supporting information.

Supporting information.

## Data Availability

Raw data and derived data supporting the findings of this study are available from the corresponding author A. R. on request.
