# Supplementary material for: The recovery trajectory of anterior cruciate ligament ruptures in randomised controlled trials: A systematic review and meta‐analysis of operative and nonoperative treatments
Source: Knee Surg Sports Traumatol Arthrosc. 2025 Feb 20;33(11):3781–93. doi: 10.1002/ksa.12626 (PMC12582240; doi:10.1002/ksa.12626)
Supplement: Supplementary file 1 — Supporting information. [file KSA-33-3781-s008.docx]

Appendix Table 1 : Characteristic of All Included studies, including those that contain the same study group.

| n | Author | Year Published | Comparison | Participants  (n) | Male  (n) | Female  (n) | Age (Mean) |
| --- | --- | --- | --- | --- | --- | --- | --- |
| 1 | Carter Et al[58] | 2023 | Reconstruction  Vs  Reconstruction | 37 | 11 | 26 | 41.886 |
| 2 | Schoepp et al [59] | 2023 | Reconstruction Vs  Reconstruction | 114 | 74 | 40 | 32.4 |
| 3 | Whang et al [60] | 2022 | Reconstruction Vs  Reconstruction | 70 | 54 | 16 | 29.1 |
| 4 | Vijay et al[61] | 2022 | Reconstruction Vs  Reconstruction | 45 | 35 | 10 | 32.7 |
| 5 | Mogos et al [62] | 2022 | Reconstruction Vs  Reconstruction | 58 | 43 | 15 | 31.2 |
| 6 | Krishna[63] | 2020 | Reconstruction Vs  Reconstruction | 56 | 43 | 13 | 27 |
| 7 | Smith et al[64] | 2020 | Reconstruction Vs  Reconstruction | 64 | 28 | 36 | 17.7 |
| 8 | Xiang et al[65] | 2019 | Reconstruction Vs  Reconstruction | 58 | 45 | 13 | 31 |
| 9 | Porter et al[66] | 2022 | Reconstruction Vs  Reconstruction | 164 | 77 | 87 | 22.7 |
| 10 | Cusumano et al[67] | 2022 | Reconstruction Vs  Reconstruction | 50 | 44 | 6 | 28.5 |
| 11 | Hoogeslag et al[68] | 2022 | Reconstruction Vs  Repair | 24 | 18 | 6 | 22 |
| 12 | Lisi et al[69] | 2022 | Reconstruction Vs  Reconstruction | 40 | 22 | 18 | 25 |
| 13 | Barnet et al[70] | 2021 | Reconstruction Vs  Reconstruction | 100 | 44 | 56 | 17 |
| 14 | Kulshrestha et al[71] | 2021 | Reconstruction Vs  Reconstruction | 80 | 80 | 0 | 30 |
| 15 | Rajput et al[42] | 2020 | Reconstruction Vs  Reconstruction | 41 | 41 | 0 | 37.2 |
| 16 | Sinding et al[30] | 2020 | Reconstruction Vs  Reconstruction | - | - | - | - |
| 17 | Von Essen et al[72] | 2020 | Reconstruction Vs  Reconstruction | 69 | 48 | 21 | 26.9 |
| 18 | Vilchez-Cavazos et al[73] | 2020 | Reconstruction Vs  Reconstruction | 28 | 23 | 5 | 23 |
| 19 | Kosters et al [74] | 2020 | Reconstruction Vs  Repair | 42 | 31 | 11 | 27.6 |
| 20 | Minguell et al[75] | 2019 | Reconstruction Vs  Reconstruction | 106 | 74 | 32 | 30.4 |
| 21 | Mohtadi et al[76] | 2019 | Reconstruction Vs  Reconstruction  Vs Reconstruction | 330 | 183 | 147 | 28.5 |
| 22 | Kouloumentas et al[77] | 2019 | Reconstruction Vs  Reconstruction | 90 | 55 | 35 | 28.7 |
| 23 | Aga et al[78] | 2018 | Reconstruction Vs  Reconstruction | 116 | 88 | 28 | 27.2 |
| 24 | Stanczak et al*[34] | 2018 | Reconstruction Vs  Reconstruction | 96 | 48 | 48 | 31.64 |
| 25 | Elveos et al [79] | 2018 | Reconstruction Vs  Reconstruction | 100 | 45 | 55 | 26 |
| 26 | Schliemann et al[80] | 2018 | Reconstruction Vs  Reconstruction | 60 | 30 | 30 | 28.7 |
| 27 | Gupta et al [32] | 2017 | Reconstruction Vs  Reconstruction | 110 | na | na | 27.1 |
| 28 | Wang et al [81] | 2017 | Reconstruction Vs  Reconstruction | 17 | 12 | 5 | 26.3 |
| 29 | Carulli et al*[41] | 2017 | Reconstruction Vs  Reconstruction | 90 | 69 | 21 | 31.4 |
| 30 | Khare et al[82] | 2017 | Reconstruction Vs  Reconstruction | 50 | 50 | 0 | 27.7 |
| 31 | Yang et al[83] | 2017 | Reconstruction Vs  Reconstruction | 60 | 34 | 26 | 34.85 |
| 32 | Karikas et al[84] | 2016 | Reconstruction Vs  Reconstruction | 103 | 70 | 33 |  |
| 33 | Chen et al[85] | 2015 | Reconstruction Vs  Reconstruction | 55 | 26 | 29 | 30.7 |
| 34 | Wang et al [86] | 2015 | Reconstruction Vs  Reconstruction | 53 | 42 | 11 | 27 |
| 35 | Zhang et al*[35] | 2014 | Reconstruction Vs  Reconstruction | 108 | 65 | 43 | 31 |
| 36 | Petersen et al[87] | 2014 | Reconstruction Vs  Reconstruction | 201 | 117 | 84 | 27 |
| 37 | Eajazi et al[88] | 2013 | Reconstruction Vs  Reconstruction vs Reconstruction | 96 | 93 | 3 | 25.5 |
| 38 | Gifstad et al[36] | 2013 | Reconstruction Vs  Reconstruction | 114 | 72 | 42 | 27 |
| 39 | Drogset et al [89] | 2005 | Reconstruction Vs  Reconstruction | 41 | 19 | 22 | 26 |
| 40 | Filbay et al [90] | 2023 | Reconstruction Vs  Rehab +/- optional Reconstruction | - | - | - | - |
| 41 | Jin et al[91] | 2022 | Reconstruction Vs  Reconstruction | 65 | 39 | 26 | 29.07 |
| 42 | Forsythe et al **[31] | 2022 | Reconstruction Vs  Reconstruction | 73 | - | - | - |
| 43 | Chahal et al[92] | 2022 | Reconstruction Vs  Reconstruction | 169 | 120 | 49 | 28.5 |
| 44 | Von Essen et al[93] | 2021 | Reconstruction Vs  Reconstruction | 137 | 69 | 68 | 33.1 |
| 45 | Mayr et al[94] | 2020 | Reconstruction Vs  Reconstruction | 30 | 21 | 9 | 26.9 |
| 46 | Jarvela et al [95] | 2017 | Reconstruction Vs  Reconstruction | 90 | 61 | 29 | 32.33 |
| 47 | Akelman et al[96] | 2016 | Reconstruction Vs  Reconstruction | 90 | 42 | 48 | 23.5 |
| 48 | Kautzner et al[97] | 2015 | Reconstruction Vs  Reconstruction | 150 | 0 | 150 | 26 |
| 49 | Lund et al[98] | 2014 | Reconstruction Vs  Reconstruction | 51 | 42 | 9 | 30.5 |
| 50 | Hohmann et al[37] | 2011 | Reconstruction Vs  Reconstruction | 40 | 30 | 10 | 27.5 |
| 51 | Nau et al[99] | 2002 | Reconstruction Vs  Reconstruction | 53 | 36 | 17 | 30.9 |
| 52 | Barenius et al[100] | 2010 | Reconstruction Vs  Reconstruction | 153 | 89 | 64 | 34 |
| 53 | Lin et al[101] | 2022 | Reconstruction Vs  Reconstruction | 51 | 45 | 6 | 30.6 |
| 54 | Beynonn et al[102] | 2011 | Reconstruction Vs  Reconstruction | 36 | 22 | 14 | 29.9 |
| 55 | Zhang et al*[103] | 2022 | Reconstruction Vs  Reconstruction | 37 | 25 | 12 | 30.4 |
| 56 | Murray et al[104] | 2020 | Reconstruction Vs  Reconstruction | - | - | - | - |
| 57 | Getgood et al[29] | 2020 | Reconstruction Vs  Reconstruction | - | - | - | - |
| 58 | Reijman et al[43] | 2021 | Reconstruction Vs  Rehab +/- optional Reconstruction | 167 | 100 | 67 | 31.3 |
| 59 | Lindstrom et al [28] | 2015 | Reconstruction Vs  Reconstruction | - | - | - | - |
| 60 | Frobell et al[105] | 2010 | Reconstruction Vs  Rehab +/- optional Reconstruction | 121 | 89 | 32 | 26.1 |
| 61 | Gifstad et al [38] | 2014 | Reconstruction Vs  Reconstruction | 110 | 71 | 39 | 24 |
| 62 | Von Essen et al[106] | 2020 | Reconstruction Vs  Reconstruction | - | - | - | - |
| 63 | Aglietti et al[39] | 2010 | Reconstruction Vs  Reconstruction | 70 | 53 | 17 | 28 |
| 64 | Gerber et al[40] | 2009 | Reconstruction Vs  Reconstruction | 40 | 24 | 16 | 29.3 |
| 65 | Birmingham et al[107] | 2008 | Reconstruction Vs  Reconstruction | 150 | 73 | 77 | 27.5 |
| 66 | Mayr et al [33] | 2010 | Reconstruction Vs  Reconstruction | 73 | - | - | 36.1 |
| 67 | Thomee et al[108] | ??? | Rehab vs Rehab | 37 | 19 | 18 | 30 |
| 68 | Irrgang et al[109] | 2021 | Reconstruction Vs  Reconstruction | 57 | 38 | 19 | 21.7 |
| 69 | Forbell et al[110] | 2013 | Reconstruction Vs  Rehab +/- optional Reconstruction | - | - | - | - |
| 70 | Sonnery-cottet et al[111] | 2020 | Reconstruction Vs  Reconstruction | 224 | 174 | 50 | 25.3 |
| 71 | Beynnon et al[112] | 2005 | Reconstruction Vs  Reconstruction | 22 | 11 | 11 | 32.55 |
| 72 | Ahlden et al[113] | 2013 | Reconstruction Vs  Reconstruction | 103 | 70 | 33 | 27.058252 |
| 73 | Mutsuazaki et al[114] | 2018 | Reconstruction Vs  Reconstruction | 90 | 47 | 43 | 25 |
| 74 | Fleming et al[115] | 2013 | Reconstruction Vs  Reconstruction | - | - | - | - |
| 75 | Panagopopulas[116] | 2021 | Reconstruction Vs  Reconstruction | 48 | 48 | 0 | 27.5 |
| 76 | Grassi et al[117] | 2021 | Reconstruction Vs  Reconstruction | 20 | 18 | 2 | 23.75 |
| 77 | Hoogeslag et al[118] | 2019 | Reconstruction Vs  Repair | - | - | - | - |
| 78 | Drogset et al[119] | 2002 | Reconstruction Vs  Reconstruction | - | - | - | - |
| 79 | Grøntvedt et al[120] | 1996 | Reconstruction Vs  Reconstruction | - | - | - | - |
| 80 | Beard et al[6] | 2022 | Reconstruction Vs  Rehab | 315 | 208 | 107 | 32.9 |
| 81 | Zhang et al | 2020 | Reconstruction Vs  Reconstruction | - | - | - | - |
| 82 | Macdonald et al | 2018 | Reconstruction Vs  Reconstruction | 88 | 58 | 30 | 31.51 |
| 83 | Grunau et al | 2016 | Reconstruction Vs  Reconstruction | 113 | - | - |  |
| 84 | McRae et al | 2013 | Reconstruction Vs  Reconstruction | 95 | 60 | 35 | 29.29 |

* Indicated medians used as age

** Numbers don’t add up on original paper

3 study demographics where not extracted as author was not able to provide the necessary PROM data to be included in the graphical or meta-analysis [28-30]

The following are part of the Same study groups

- 77 and 11
- 13 and 56
- 25, 78, and 79
- 47 and 74
- 55 and 81
- 69, 60 and 40
- 17 and 62
