# Supplementary figures and images for: The recovery trajectory of anterior cruciate ligament ruptures in randomised controlled trials: A systematic review and meta‐analysis of operative and nonoperative treatments
Source: Knee Surg Sports Traumatol Arthrosc. 2025 Feb 20;33(11):3781–93. doi: 10.1002/ksa.12626 (PMC12582240; doi:10.1002/ksa.12626)

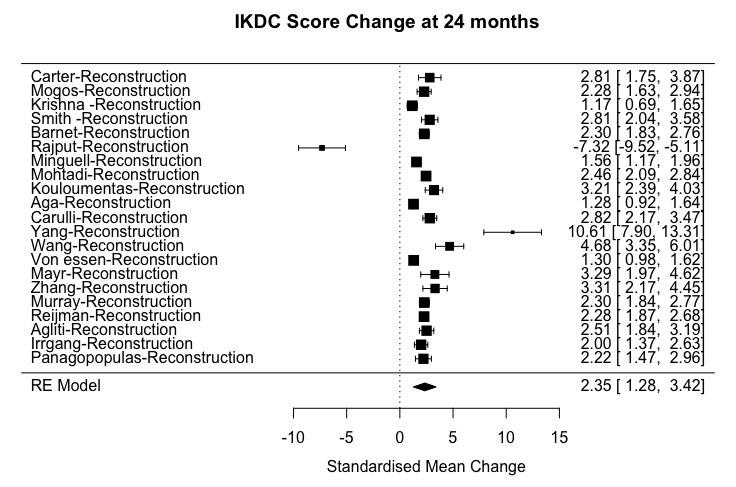

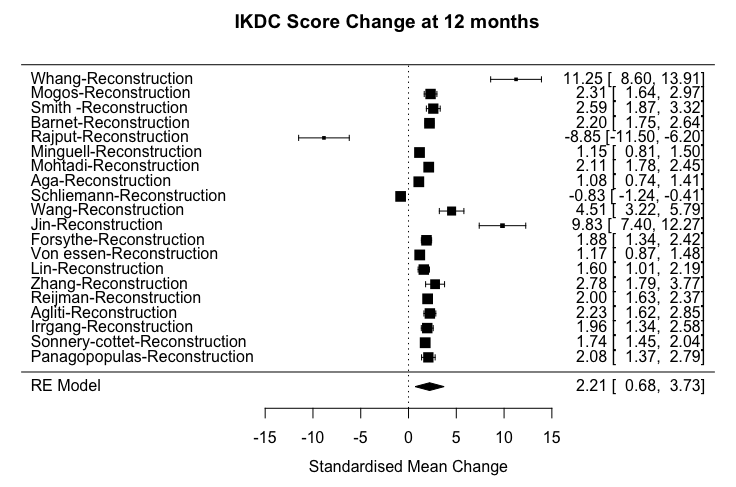

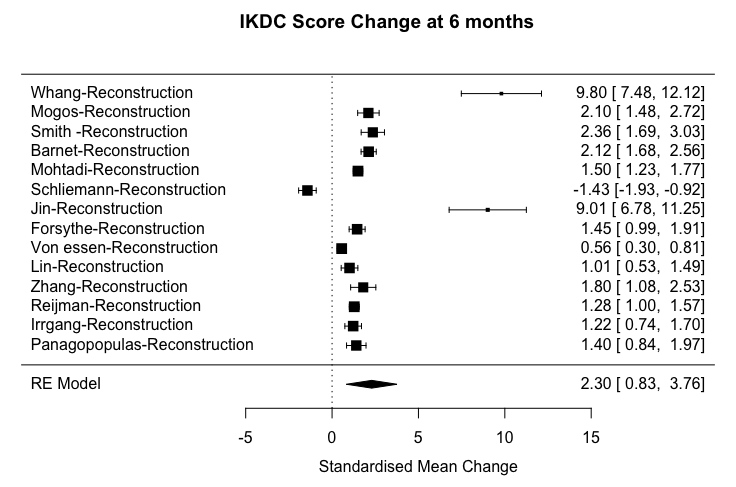

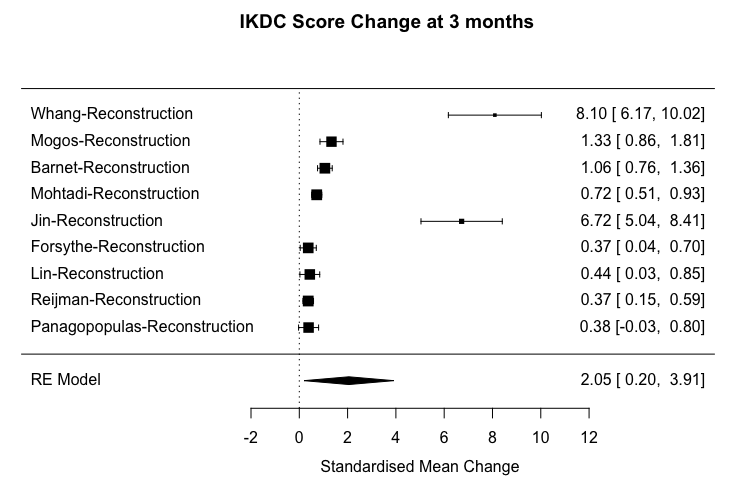
Supplementary Material 4: IKDC Forest plot without the rehabilitation study.

Supplement: Supplementary file 2 — Supporting information. [file KSA-33-3781-s017.docx]

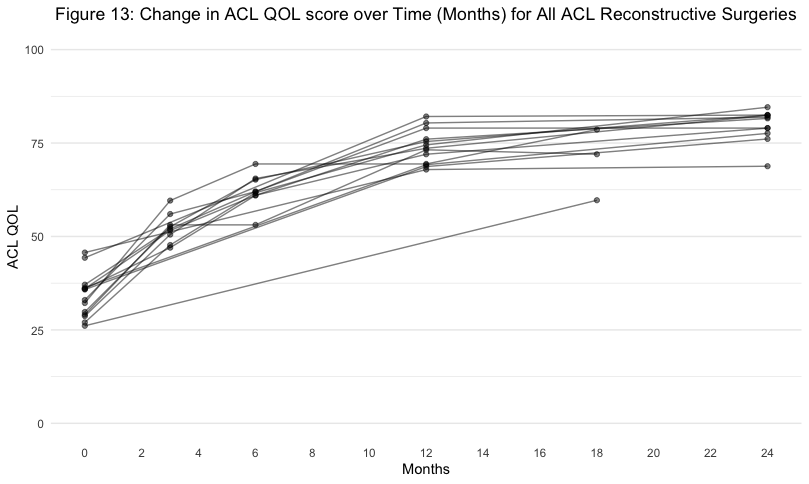

Supplement: Supplementary file 5 — Supporting information. [file KSA-33-3781-s007.png]

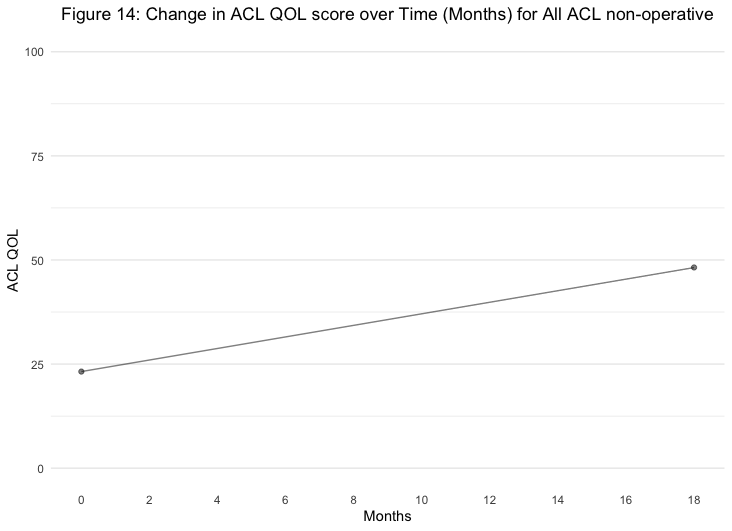

Supplement: Supplementary file 7 — Supporting information. [file KSA-33-3781-s013.png]

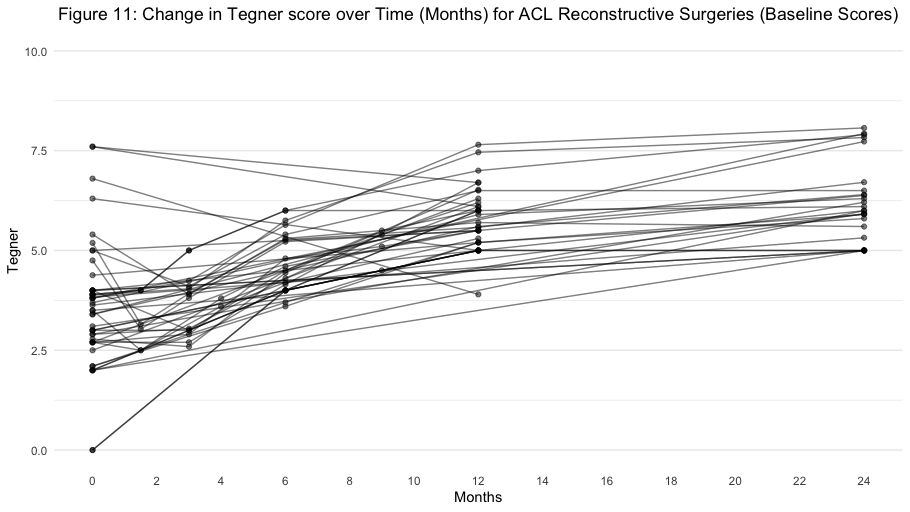

Supplement: Supplementary file 8 — Supporting information. [file KSA-33-3781-s003.png]

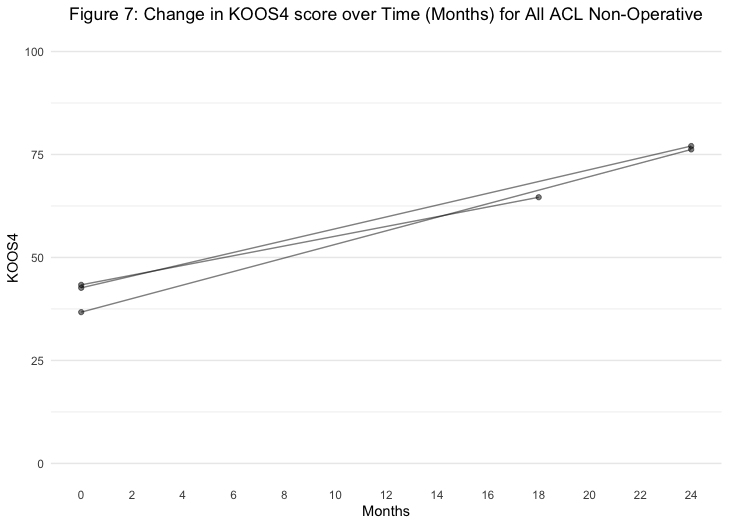

Supplement: Supplementary file 10 — Supporting information. [file KSA-33-3781-s014.png]

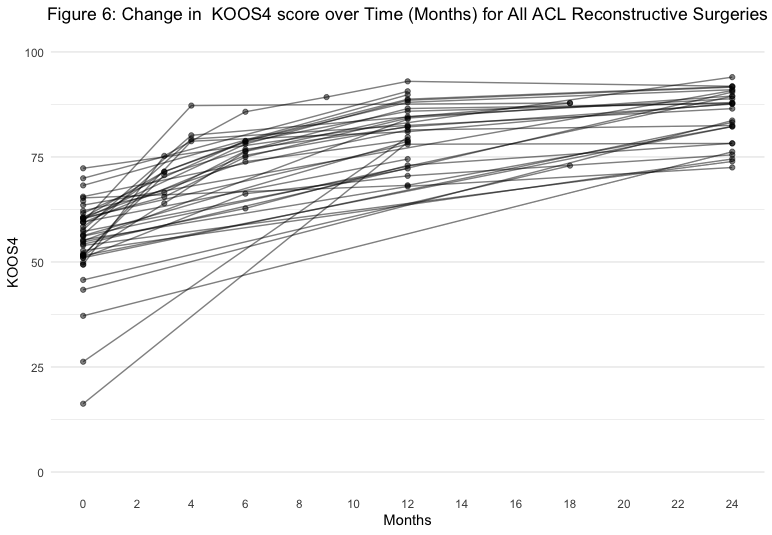

Supplement: Supplementary file 11 — Supporting information. [file KSA-33-3781-s005.png]

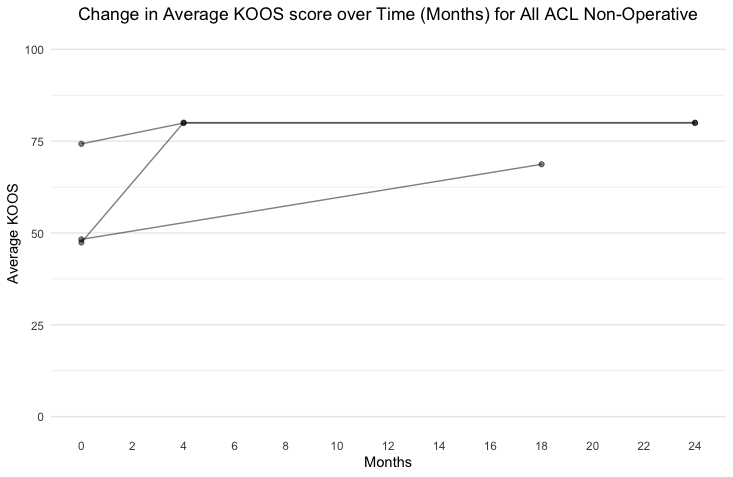

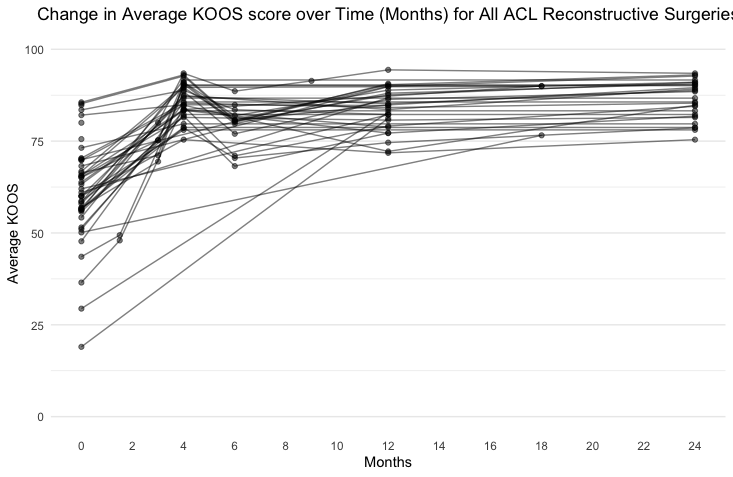

Supplement: Supplementary file 12 — Supporting information. [file KSA-33-3781-s004.docx]

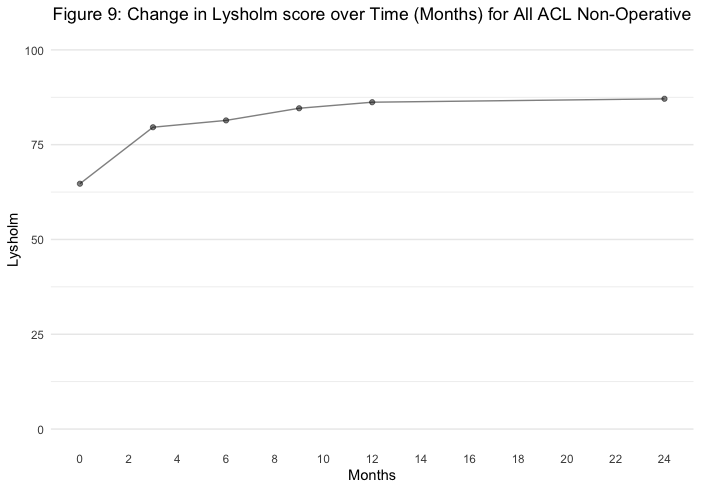

Supplement: Supplementary file 13 — Supporting information. [file KSA-33-3781-s001.png]

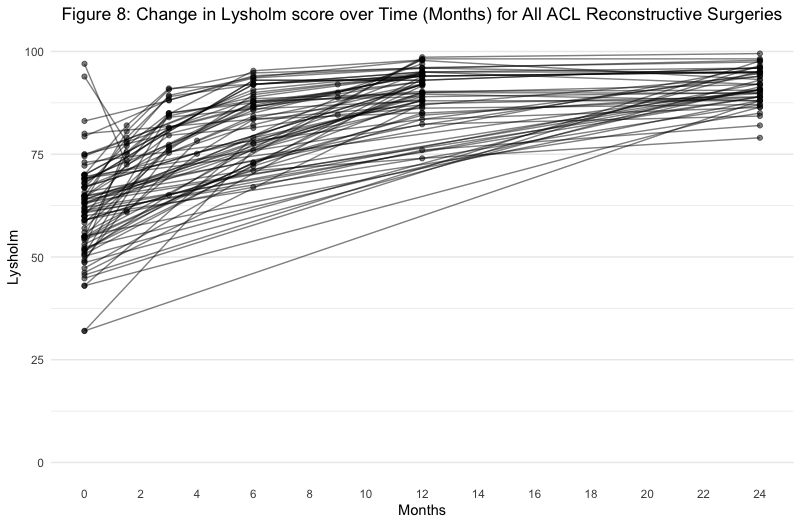

Supplement: Supplementary file 14 — Supporting information. [file KSA-33-3781-s010.png]

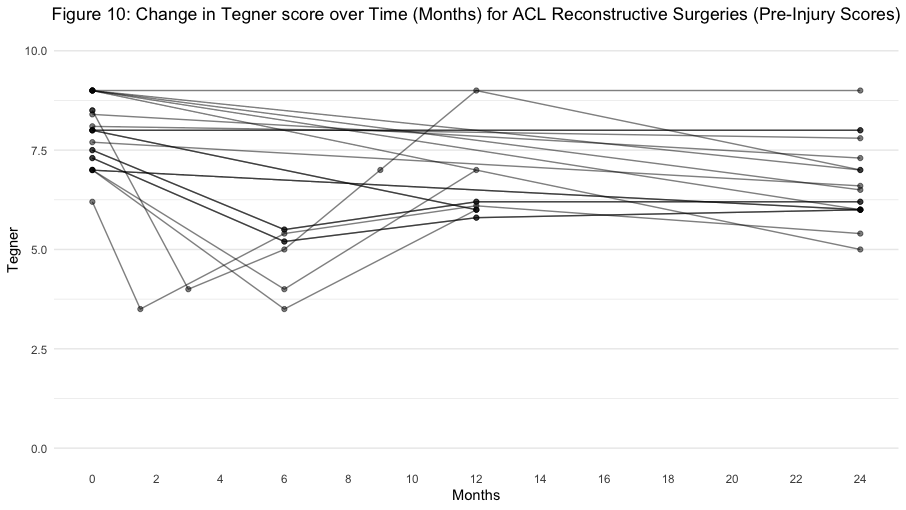

Supplement: Supplementary file 15 — Supporting information. [file KSA-33-3781-s009.png]

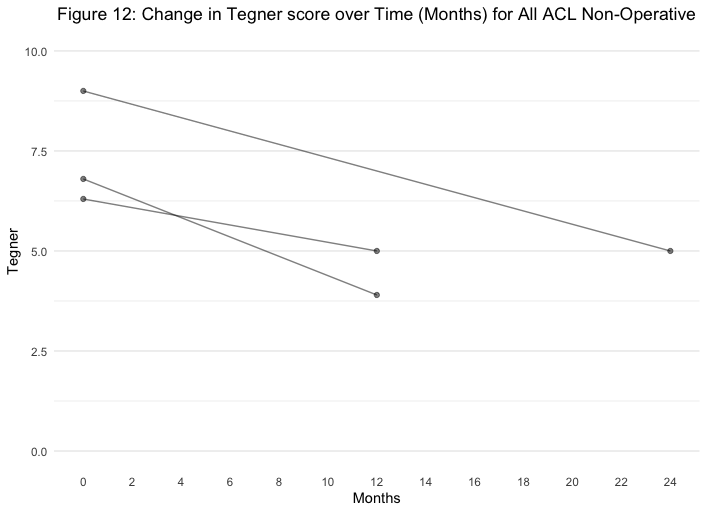

Supplement: Supplementary file 16 — Supporting information. [file KSA-33-3781-s002.png]

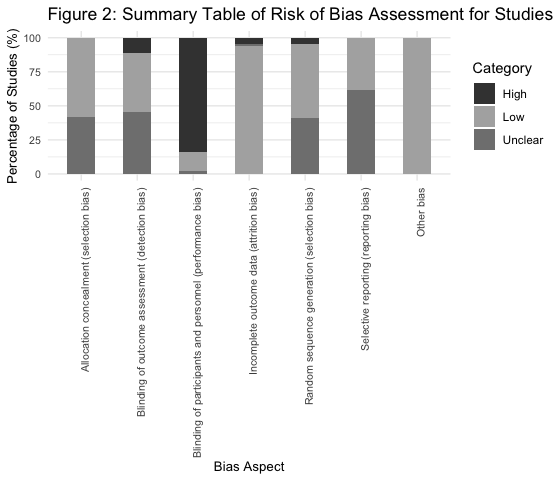

Supplement: Supplementary file 17 — Supporting information. [file KSA-33-3781-s012.png]
