# Supplementary material for: The recovery trajectory of anterior cruciate ligament ruptures in randomised controlled trials: A systematic review and meta‐analysis of operative and nonoperative treatments
Source: Knee Surg Sports Traumatol Arthrosc. 2025 Feb 20;33(11):3781–93. doi: 10.1002/ksa.12626 (PMC12582240; doi:10.1002/ksa.12626)
Supplement: Supplementary file 3 — Supporting information. [file KSA-33-3781-s011.docx]

Supplementary Table 2

| PROM | Number of studies that used PROM |
| --- | --- |
| IKDC score | 32 |
| KOOS score | 24 |
| Lysholm | 42 |
| Tegner | 38 |
| Cincinnati Knee Rating System (CKRS) or Modified version. | 2 |
| ACL QOL | 7 |
