# Supplementary material for: The recovery trajectory of anterior cruciate ligament ruptures in randomised controlled trials: A systematic review and meta‐analysis of operative and nonoperative treatments
Source: Knee Surg Sports Traumatol Arthrosc. 2025 Feb 20;33(11):3781–93. doi: 10.1002/ksa.12626 (PMC12582240; doi:10.1002/ksa.12626)
Supplement: Supplementary file 4 — Supporting information. [file KSA-33-3781-s006.docx]

Table 1: *Authors contacted for study and who responded*

| Author | Paper | Reason for contacting | Responded |
| --- | --- | --- | --- |
| Schoepp et all 2023 | Brace-Free Rehabilitation after Isolated Anterior Cruciate Ligament Reconstruction with Hamstring Tendon Autograft Is Not Inferior to Brace-Based Rehabilitation-A Randomised Controlled Trial | Use of MCID for IKDC scores | N |
| Lisi et al 2022 | The effect of proprioception training on knee kinematics after anterior cruciate ligament reconstruction: A randomized control trial | Baseline Lysholm Scores not included | N |
| Rajput et al 2020 | To compare the functional outcome of the Patellar Tendon and hamstring tendon autograft for anterior cruciate ligament reconstruction in males | Low IKDC scores (in the single digits) emailed to clarify if this was in error | N |
| Sinding et al 2020 | Effects of Autograft Types on Muscle Strength and Functional Capacity in Patients Having Anterior Cruciate Ligament Reconstruction: A Randomized Controlled Trial | Figures for IKDC mentioned in study but not provided | N |
| Von Essen et al 2020 | Acute reconstruction results in less sick-leave days and as such fewer indirect costs to the individual and society compared to delayed reconstruction for ACL injuries | KOOS scores estimated from graphical representation. Emailed for their exact figures and means | N |
| Kosters et al 2020 | Repair With Dynamic Intraligamentary Stabilization Versus Primary Reconstruction of Acute Anterior Cruciate Ligament Tears: 2-Year Results From a Prospective Randomized Study | Tegner scores estimated from graphical representation | N |
| Elveos et al 2018 | Anterior Cruciate Ligament Reconstruction Using a Bone-Patellar Tendon-Bone Graft With and Without a Ligament Augmentation Device: A 25-Year Follow-up of a Prospective Randomized Controlled Trial | Lysholm and Tegner scores estimated from graphical represenatations | N |
| Khare et al 2017 | Randomised comparison of pretensioning using cyclical loading and on tendon board for arthroscopic anterior cruciate ligament reconstruction using hamstring autograft | Means provided for PROMS but no SD | N |
| Peterson et al 2014 | Long-term results of a randomized study on anterior cruciate ligament reconstruction with or without a synthetic degradable augmentation device to support the autograft | KOOS scores estimated from graphical representation. Figures requested and SD requested | N |
| Von Essen et al 2021 | Utilizing a contralateral hamstring autograft facilitates earlier isokinetic and isometric strength recovery after anterior cruciate ligament reconstruction: a randomised controlled trial. | KOOS scores estimated from graphical representation. Figures requested along with SD | Y |
| Nau 2002 | A new generation of artificial ligaments in reconstruction of the anterior cruciate ligament. Two-year follow-up of a randomised trial. | KOOS scores estimated from graphical representation. Figures requested along with SD | N |
| Getgood et al 2020 | Lateral Extra-articular Tenodesis Reduces Failure of Hamstring Tendon Autograft Anterior Cruciate Ligament Reconstruction: 2-Year Outcomes From the STABILITY Study Randomized Clinical Trial | No Baseline figures for PROMs provided in study. However, these are mentioned in the study. Emailed for these | N |
| Lindstr√∂m et al 2015 | Post-operative bracing after ACL reconstruction has no effect on knee joint effusion. A prospective, randomized study | PROMS provided as Odds ratio. Requested means and SD. | N |
| Gifstad et al 2014 | Femoral fixation of hamstring tendon grafts in ACL reconstructions: the 2-year follow-up results of a prospective randomized controlled study | KOOS scores estimated from graphical representation. Figures requested along with SD | N |
| Mayr et al 2010 | Rehabilitation results following anterior cruciate ligament reconstruction using a hard brace compared to a fluid-filled soft brace | IKDC scores estimated from graphical representation. Figures requested along with SD. | N |
| Irrgang et al 2021 | Anatomic single vs. double-bundle ACL reconstruction: a randomized clinical trial-Part 1: clinical outcomes | IKDC scores estimated from graphical representation. Figures requested along with SD. | Y |
| Sonnery-Cottet et al 2020 | Combined ACL and Anterolateral Reconstruction Is Not Associated With a Higher Risk of Adverse Outcomes: preliminary Results From the SANTI Randomized Controlled Trial | KOOS scores estimated from graphical representation. Figures requested along with SD | N |
| Reijman et al 2021 | Early surgical reconstruction versus rehabilitation with elective delayed reconstruction for patients with anterior cruciate ligament rupture: COMPARE randomised controlled trial | Clarification if any patients with collateral ligament injury underwent surgical operation | Y |
| Grassi et al 2021 | Hamstring grafts for anterior cruciate ligament reconstruction show better magnetic resonance features when tibial insertion is preserved | KOOS scores estimated from graphical representation. Figures requested along with SD | Y |
| Macdonald et al 2018 | Anteromedial portal vs Transtibial technique in ACL reconstruction | Scores for ACL QOL are estimated from graphical representation | N |
