# Supplementary material for: The recovery trajectory of anterior cruciate ligament ruptures in randomised controlled trials: A systematic review and meta‐analysis of operative and nonoperative treatments
Source: Knee Surg Sports Traumatol Arthrosc. 2025 Feb 20;33(11):3781–93. doi: 10.1002/ksa.12626 (PMC12582240; doi:10.1002/ksa.12626)
Supplement: Supplementary file 9 — Supporting information. [file KSA-33-3781-s016.docx]

| KOOS ADL | |
| --- | --- |
| 3 Months | 0.46 (95% CI: -0.11,1.03) |
| 6 Months | 0.99 (95% CI: 0.34,1.65) |
| 12 Months | 0.99 (95% CI: 0.53,1.45) |
| 18 Months (ACL SNAPP Reconstruction) | 1.02 (95% CI: 0.83,1.21) |
| 18 Months (ACL SNAPP Rehab) | 0.80 (95% CI: 0.62,0.98) |
| 24 Months (Pooled) | 1.18 (95% CI:0.67,1.68) |
| 24 Months (Reconstruction Group only) | 1.22 (95% CI: 0.57,1.87) |
| 24 Months (Rehab Group Only) | 0.97 (95% CI: 0.39,1.55) |
| 24 Months (Optional Reconstruction Group) | 1.31 (95% CI: 0.75,1.87) |
| KOOS Pain | |
| 3 Months | 0.87 (95% CI: 0.57,1.17) |
| 6 Months | 1.22 (95% CI: 0.33,2.12) |
| 12 Months | 1.29 (95% CI: 0.52,2.05) |
| 18 Months (ACL SNAPP Reconstruction) | 1.45 (95% CI: 1.22,1.67) |
| 18 Months (ACL SNAPP Rehab) | 1.11 (95% CI: 0.91,1.30) |
| 24 Months (Pooled) | 1.34 (95% CI: 0.86,1.82) |
| 24 Months (Reconstruction Group only) | 1.35 (95% CI: 0.74,1.97) |
| 24 Months (Rehab Group Only) | 1.25 (95% CI:0.34,2.16) |
| 24 Months (Optional Reconstruction Group) | 1.61 (95% CI: 0.99,2.23) |
| KOOS QOL | |
| 3 Months | 0.69 (95% CI: 0.23,1.14) |
| 6 Months | 1.90 (95% CI: 0.71,3.09) |
| 12 Months | 2.35 (95% CI: 1.21, 3.49) |
| 18 Months (ACL SNAPP Reconstruction) | 1.45 (95% CI: 1.22, 1.67) |
| 18 Months (ACL SNAPP Rehab) | 1.11 (95% CI: 0.91, 1.30) |
| 24 Months (Pooled) | 2.16 (95% CI:1.75, 2.57) |
| 24 Months (Reconstruction Group only) | 2.19 (95% CI: 1.67,2.71) |
| 24 Months (Rehab Group Only) | 1.97 (95% CI:1.66,2.28) |
| 24 Months (Optional Reconstruction Group) | 2.36 (95% CI:1.57,3.16) |
| KOOS Sport | |
| 3 Months | 0.61 (95% CI: 0.30,0.92) |
| 6 Months | 1.36 (95% CI: 0.59,2.14) |
| 12 Months | 1.70 (95% CI: 1.00,2.40) |
| 18 Months (ACL SNAPP Reconstruction) | 1.26 (95% CI: 1.05,1.47) |
| 18 Months (ACL SNAPP Rehab) | 0.97 (95% CI: 0.78,2.40) |
| 24 Months (Pooled) | 2.04 (95% CI: 1.49, 2.60) |
| 24 Months (Reconstruction Group only) | 1.93 (95% CI: 1.29,2.57) |
| 24 Months (Rehab Group Only) | 2.36 (95% CI: 0.91,3.82) |
| 24 Months (Optional Reconstruction Group) | 3.12 (95% CI: 2.13,4.11) |
| KOOS Symptom | |
| 3 Months | 0.48 (95% CI: 0.20,0.75) |
| 6 Months | 1.18 (95% CI: 0.61,1.76) |
| 12 Months | 1.23 (95% CI: 0.62,1.84) |
| 18 Months (ACL SNAPP Reconstruction) | 1.02 (95% CI: 0.82,1.21) |
| 18 Months (ACL SNAPP Rehab) | 0.91 (95% CI: 0.72,1.09) |
| 24 Months (Pooled) | 1.61 (95% CI: 0.78,2.44) |
| 24 Months (Reconstruction Group only) | 1.61 (95% CI: 0.55 ,2.67) |
| 24 Months (Rehab Group Only) | 1.57 (95% CI: 0.46,2.69) |
| 24 Months (Optional Reconstruction Group) | 2.20 (95% CI:1.45,2.96) |

Full Table for KOOS subscales

**Table 1:** *Table showing the SMC changes for each KOOS subscale at 3,6,12 and 24 months along with their respective 95% confidence interval.* *ACL SNAPP 18 month SMC is also included.*
